# Dental service utilization in the very old: an insurance database analysis from northeast Germany

**DOI:** 10.1007/s00784-020-03591-z

**Published:** 2020-09-30

**Authors:** Falk Schwendicke, Aleksander Krasowski, Jesus Gomez Rossi, Sebastian Paris, Adelheid Kuhlmey, Hendrik Meyer-Lückel, Joachim Krois

**Affiliations:** 1 Department of Oral Diagnostics, Digital Health and Health Services Research, Charité – Universitätsmedizin Berlin, corporate member of Freie Universität Berlin, Humboldt-Universität zu Berlin, and Berlin Institute of Health, Aßmannshauser Str., 4-6, 14197 Berlin, Germany; 2 Department of Restorative, Preventive and Pediatric Dentistry, zmk bern, University of Bern, Bern, Switzerland; 3 Department of Operative and Preventive Dentistry, Charité – Universitätsmedizin Berlin, corporate member of Freie Universität Berlin, Humboldt-Universität zu Berlin, and Berlin Institute of Health, Berlin, Germany; 4 Institute of Medical Sociology and Rehabilitation Science, Charité – Universitätsmedizin, Berlin, corporate member of Freie Universität Berlin, Humboldt-Universität zu Berlin, and Berlin Institute of Health, Berlin, Germany

**Keywords:** Access, Geriatrics, Gerodontology, Health services research, Operative dentistry, Surgery

## Abstract

**Objectives:**

We assessed dental service utilization in very old Germans.

**Methods:**

A comprehensive sample of 404,610 very old (≥ 75 years), insured at a large statutory insurer (Allgemeine Ortskrankenkasse Nordost, active in the federal states Berlin, Brandenburg, Mecklenburg-Western Pomerania), was followed over 6 years (2012–2017). Our outcome was the utilization of dental services, in total (any utilization) and in five subgroups: (1) examinations and associated assessment or advice, (2) restorations, (3) surgery, (4) prevention, (5) outreach care. Association of utilization with (1) sex, (2) age, (3) region, (4) social hardship status, (5) ICD-10 diagnoses, and (6) German modified diagnosis-related groups (GM-DRGs) was explored.

**Results:**

The mean (SD) age of the sample was 81.9 (5.4) years. The utilization of any dental service was 73%; utilization was highest for examinations (68%), followed by prevention (44%), surgery (33%), restorations (32%), and outreach care (13%). Utilization decreased with age for nearly all services except outreach care. Service utilization was significantly higher in Berlin and most cities compared with rural municipalities, and in individuals with common, less severe, and short-term conditions compared with life-threatening and long-term conditions. In multi-variable analysis, social hardship status (OR: 1.14; 95% CI: 1.12-1.16), federal state (Brandenburg 0.85; 0.84–0.87; Mecklenburg-Western Pomerania: 0.80; 0.78–0.82), and age significantly affected utilization (0.95; 0.95–0.95/year), together with a range of co-morbidities according to ICD-10 and DRG.

**Conclusions:**

Social, demographic, regional, and general health aspects were associated with the utilization of dental services in very old Germans. Policies to maintain access to services up to high age are needed.

**Clinical significance:**

The utilization of dental services in the very old in northeast Germany showed significant disparities within populations. Policies to allow service utilization for sick, economically disadvantaged, rural and very old populations are required. These may include incentives for outreach servicing, treatment-fee increases for specific populations, or referral schemes between general medical practitioners and dentists.

**Electronic supplementary material:**

The online version of this article (10.1007/s00784-020-03591-z) contains supplementary material, which is available to authorized users.

## Introduction

For decades, interventions to improve dental health have been focused on children and adolescents, with widely acknowledged success in many high-income countries. While adults and older individuals also benefitted from a general improvement in oral health, showing a reduced number of restored or missing teeth [1, 2], data on the resulting treatment needs in these populations are scarce. Especially for the very old, defined as those aged 75 years or older, there is very limited knowledge on their needs for and utilization of dental service. This group of very old, notably, is the only growing one in many high-income countries, with remarkably complex oral health dynamics: Retaining an increasing number of teeth up to such high age, this group is, oftentimes suddenly, affected by general health deterioration, impacting on the capability for oral self-care as well as the physical abilities to utilize in-office dental care [3–6].

In a previous study and building on claims data, we found a disparate utilization of prosthetic services in the very old, with those aged 85 years or older, those living rural, and those with severe general health conditions utilizing prosthetic services, by large, to a lower degree than younger, urban living and only limitedly sick seniors [7]. The only service the former group used more often was maintenance of existing prosthetics. Notably**,** claims data come with a range of possible limitations, e.g., selection bias, confounding bias, or misclassification bias. However, employing claims data allows to investigate groups which are otherwise hard to represent, e.g., the very old, the sick, and the rural living ones. Claims data also come with robust sample sizes and represent everyday care. They also suffer from limited risks of recollection or reporting bias and have a high generalizability for their respective healthcare setting [8, 9].

In the present study, we used claims data from a large health insurance in northeast Germany to assess dental service utilization in the very old. We hypothesized that utilization differed according to age, general health, socioeconomic status, and place of living.

## Methods

### Study design

For reasons of comparability, the design and conduct of this study largely aligns with that of a previous publication on prosthetic treatment patterns in the same population [7]. The investigated cohort was evaluated based on routinely collected claims data from a statutory (public) health insurance in Germany. Individuals aged 75 years or older from one large insurer, the AOK Nordost, were followed over 6 years (2012 to 2017). The AOK Nordost is a regional branch of the Allgemeine Ortskrankenkasse (AOK), acting mainly in the Northeast of Germany in the federal states of Berlin, Brandenburg, and Mecklenburg-Western Pomerania. Our reporting follows the RECORD statement [10].

### Setting

The AOK Nordost insures around 1.8 million individuals from the described three federal states. Insured individuals may, however, also move into other areas of Germany, which is why for our geographic analyses only individuals living in these federal states between 2012 and 2017 were included. The area of interest encompasses the German capital, Berlin, and two rural states, Brandenburg and Mecklenburg-Western Pomerania, with only few larger cities (> 70,000 inhabitants). All three states are considered economically weak in comparison with other parts of Germany.

Data for this study were claims data, including claims from 1 January 2012 to 31 December 2017. Data were routinely collected and provided under ethical approval in a pseudonymized form using a data protection cleared platform via the scientific institute of the AOK Nordost, the GEWiNO.

### Participants and sample size

A comprehensive sample of very old, aged 75 years or above, insured with the AOK Nordost in 2012, was drawn and followed over 6 years. No further eligibility criteria were defined. Variable ascertainment was only possible via insurance base data and claims data. The database had been curated for plausibility at GEWiNO and once more by the study team. No formal sample size estimation was performed given this being a comprehensive sample.

### Variables

Our outcome was the relative utilization (in % of the population) of dental services. Within the statutory German insurance, dental services are provided on a fee-per-item basis using fee items catalogs of the statutory or private German insurance [11, 12]. The vast majority (88%) of patients are statutorily insured. For the statutory insurance, all items are drawn from the fee item catalog Bewertungsmaßstab (BEMA), which contains a large range of granular items comprising (1) examinations, assessment and advice, radiographic evaluations etc. (Examinations); (2) restorative dentistry (Restorations), note that within German insurance coding, crowns are not subsumed under “restorations” and hence there is no overlap between this service group and our previous analysis on prosthetic dentistry; (3) oral surgery and medicine (Surgery); (4) Prevention (for adults, the only preventive measure available until 2015 was removal of calculus; in 2015, further fee items (focusing on oral hygiene measurement, and oral hygiene plan, denture cleaning, and fluoride application) were introduced but these were not available for the present analysis); and (5) Outreach care. Further items include, for example, periodontal treatment, prosthetic therapies, and adjunct measures. We here report on any utilization in BEMA (min 1 item claimed/year) as well as stratified along the item blocks 1–5.

As this is the first detailed analysis on dental service utilization in the very old in northeast Germany, we provide largely descriptive analyses. The utilization of dental services was assessed according to following independent variables: (1) sex (male/female); (2) age (in years) in each year of follow-up; (3) region, we used municipalities as regional units, mainly as on a lower (more granular) spatial level only few individuals were retained in some areas. Municipalities included the capital Berlin (with over 3.5 million inhabitants), medium-sized cities (70,000–200,000 inhabitants), and rural areas. Further analyses were performed on federal state level; (4) social hardship status (income < 1246 Euro/month per capita in 2019); (5) ICD-10 diagnoses, derived from outpatient diagnostic data; (6) inpatient hospital diagnoses and treatments, derived from German modified diagnosis-related groups (GM-DRGs). The GM-DRGs classify diseases in groups of similar pathogenesis, characteristics, and treatment complexity, and are mainly used for reimbursement reasons. Only the 25 most frequently recorded ICD-10 and GM-DRG codes were used.

### Data sources and access

The data used for this study were provided by the GEWiNO using a data protection approved storage and analysis platform after cleaning and consistency controls. Data were pseudonymized and included individuals’ age, sex, social hardship status, spatial code of their place of living (allowing classification into municipalities), all BEMA items claimed per year as well as ICD-10 codes and GM-DRGs for each year, among further variables. Comparability of data between different years and data consistency was given.

### Bias

A comprehensive sample had been used, and neither participants nor providers were aware that the collected claims data will be used for routine data analyses later on. The data collection is not prone to selection and detection bias. However, given this being claims data from only one insurance, the overall population of very old Germans differs and data may be affected by biases associated with claims data, as laid out above and in the discussion. No further measures against these biases could be taken.

### Statistical analyses

The statistical analysis was performed on a sample (*n* = 404,610) of the database provided by AOK Nordost. The only inclusion criterion was that an individual had to be insured in the year 2012 and had to be aged 75 years or above at this point. For the descriptive analysis of utilization of dental services, we considered an individual to have consumed a particular service if at least once during the period 2012 to 2017 the provision of such a service was claimed. Descriptive statistics of age groups were computed based on the age distribution in 2012. An individual was assigned to having a social hardship if the individual was assigned to this status at least once during the period 2012 to 2017. For geographical analysis, we excluded all individuals that relocated from one of the federal states (Berlin, Brandenburg, and Mecklenburg-Western Pomerania) to another federal state, thereby decreasing the sample size to 390,044. However, we did not correct for relocations within the three federal states during the observational period.

For each particular outpatient diagnosis (ICD-10 codes) and inpatient hospital diagnosis and treatment (GM-DRGs), we summed up all claims and ranked them from most to least frequent. We then selected the 25 most frequent diagnoses each (in total 50) and computed for each of them the number of individuals that were assigned to having a diagnosis, respectively, treatment, during 2012 to 2017.

We applied logistic regression, a method to model a binary outcome variable as a linear combination of predictor variables. The response variable was the utilization of any type of dental services claimed by an individual at least once in the year 2012. As predictor variables we included age, sex, being deceased, social hardship status, federal state (note that we allowed the category “other” for relocated individuals), and the described outpatient and inpatient hospital diagnosis variables, all of them referring to the year 2012. All analyses, modeling, and visualization were performed using Python (version 3.7, available at http://www.python.org) and auxiliary modules from its scientific computing ecosystem.

## Results

Overall, 404,610 very old (75 years or older) individuals were followed over a period of up to 6 years (173,733 of these did not survive follow-up). The mean (SD, median, min, max) age of the sample was 81.9 (5.4, 81, 75, 109) years. The population comprised significantly more females than males and those aged 75–84 years old than those aged 85 years or older. About one-third lived in Berlin, and the other two-thirds in the more rural Brandenburg and Mecklenburg-Western Pomerania. Social hardship status was claimed by nearly half of the population at least once during the follow-up period (Table 1). Our sample was overall more female and much older and claimed far more hardship status than the national average.

**Table 1:** Sample characteristics (*N*; %) from northeast Germany. Total, male, and female population aged 75 years or older, in 5-year age bands and according to federal state

Covariate	Group	*N*	Any service	Examination	Restorative	Surgical	Prevention	Outreach
All*		404,610 (100.0)	294,469 (72.8)	276,481 (68.3)	130,533 (32.3)	132,130 (32.7)	177,308 (43.8)	54,210 (13.4)
Sex	Male**	134,909 (33.3)	100,958 (74.8)	97,089 (72.0)	48,714 (36.1)	48,544 (36.0)	64,473 (47.8)	13,012 (9.6)
	Female	269,702 (66.7)	193,511 (71.7)	179,392 (66.5)	81,819 (30.3)	83,586 (31.0)	112,835 (41.8)	41,198 (15.3)
Age group***	75–79	162,368 (22.7)	122,450 (75.4)	119,971 (73.9)	58,321 (35.9)	48,261 (29.7)	83,694 (51.5)	6416 (4.0)
	80–84	266,956 (37.4)	190,529 (71.4)	182,964 (68.5)	78,514 (29.4)	68,123 (25.5)	118,148 (44.3)	17,487 (6.6)
	85–89	174,672 (24.5)	110,515 (63.3)	101,092 (57.9)	35,594 (20.4)	34,596 (19.8)	57,781 (33.1)	20,727 (11.9)
	90–94	82,597 (11.6)	44,539 (53.9)	36,290 (43.9)	9931 (12.0)	11,332 (13.7)	18,104 (21.9)	15,799 (19.1)
	95–99	22,641 (3.2)	10,117 (44.7)	6716 (29.7)	1355 (6.0)	1903 (8.4)	2977 (13.1)	5498 (24.3)
	100–104	4214 (0.6)	1598 (37.9)	855 (20.3)	103 (2.4)	185 (4.4)	306 (7.3)	1123 (26.6)
	105–109	348 (0.0)	107 (30.7)	46 (13.2)	3 (0.9)	7 (2.0)	12 (3.4)	85 (24.4)
Social hardship status	No	210,292 (52.0)	145,768 (69.3)	138,483 (65.9)	68,008 (32.3)	67,763 (32.2)	92,050 (43.8)	20,315 (9.7)
	Yes****	194,318 (48.0)	148,701 (76.5)	137,998 (71.0)	62,525 (32.2)	64,367 (33.1)	85,258 (43.9)	33,895 (17.4)
Federal state	Berlin	122,454 (30.3)	90,273 (73.7)	84,544 (69.0)	43,287 (35.3)	44,910 (36.7)	59,622 (48.7)	19,916 (16.3)
	Brandenburg	153,164 (37.9)	110,254 (72.0)	104,375 (68.1)	48,832 (31.9)	48,774 (31.8)	66,264 (43.3)	15,763 (10.3)
	Mecklenburg	107,665 (26.6)	77,969 (72.4)	72,878 (67.7)	32,040 (29.8)	31,773 (29.5)	42,415 (39.4)	13,413 (12.5)
	Others	21,327 (5.3)	15,973 (74.9)	14,684 (5.3)	6374 (29.9)	6673 (31.3)	9007 (42.2)	5118 (24.0)

National data: *83.200.000; **49.3% male; ***44.5 years (www.destatis.de); ****11% in 2018 [29]

**Fig. 1:**
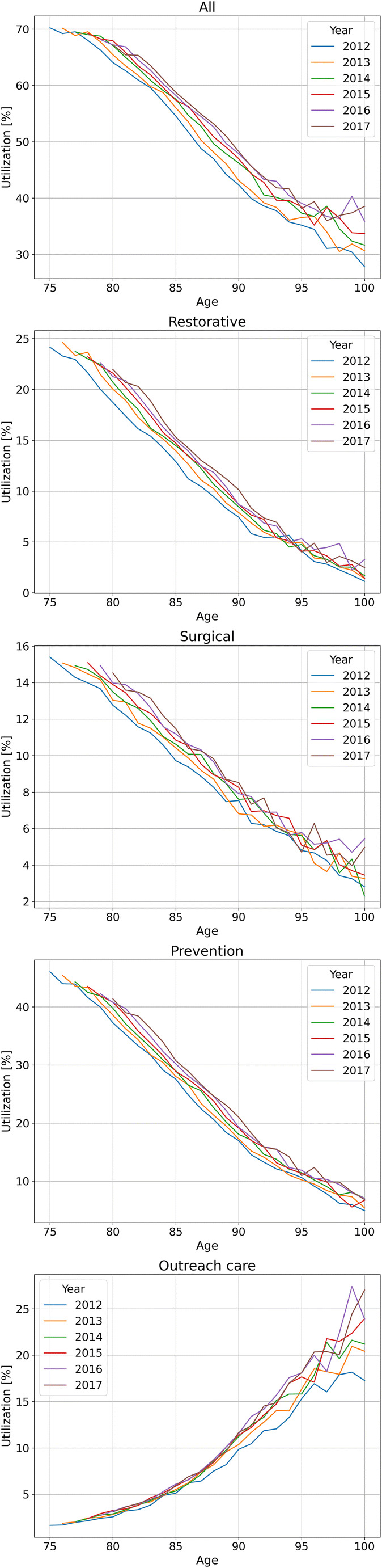
Utilization (in %) of dental services by the very old in northeast Germany. Any utilization and specific service utilization are shown. Individuals available in 2012 of all ages from 75 years upwards (blue line) were followed over 6 years until 2017 (black line); i.e., the 75-years in 2012 are the 76 years in 2013 etc. (which is why the lines start further to the right with longer follow-up)

The utilization of any dental service was 73%; utilization was highest for examinations (68%), followed by prevention (44%), surgery (33%), and restorative (32%) and outreach care (13%). Utilization decreased with age for nearly all services except outreach care (Fig. 1). Utilization of restorations, surgery, and prevention decreased by 75–80% (in relative terms, e.g., from 36% to 6% for restorations) between age 75 and 95 years; the decrease after age 95 years was limited. A slightly less pronounced, but nevertheless consistent, decrease was found for examinations. In contrast, outreach care increased and was, at age 95 years or above, the main service (together with examinations, which one would assume is the minimum consequence of outreach care).

**Fig. 2:**
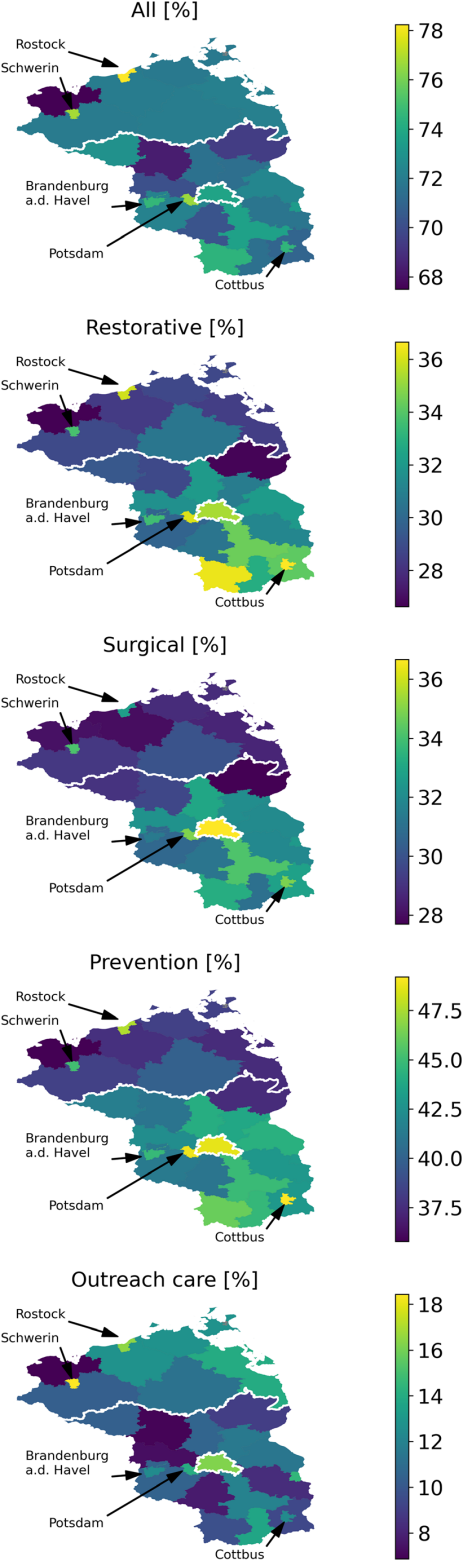
Regionally specific utilization of dental services, stratified in services blocks, in northeast Germany. Relative (in %) any utilization and specific service utilization is shown. Larger cities with an increased or decreased utilization compared with the surrounding municipalities are further highlighted by arrows

Utilization was further different between regions (Table 1, Fig. 2). Utilization of any dental service was generally higher in cities than rural areas, and highest in Berlin and three other urban municipalities (Rostock, Potsdam, Schwerin). Utilization further differed geographically according to specific services. Utilization of restorations was nearly 50% increased in certain cities and one rural Southwestern municipality compared with most other rural areas. Surgical services were provided more often in Berlin and the South as well as cities in general; a similar pattern was observed for preventive services. For outreach care, no such strict pattern was observed; certain cities as well as a stretch of municipalities along the coastline showed higher utilization.

Utilization of any dental service was assessed according to ICD-10 codes (Table 2). Utilization was higher for the majority of codes, e.g., for eye conditions (e.g., presbyopia, cataract, astigmatism), gonarthrosis, cox-arthrosis, benign hypertension, anti-coagulants therapy, varicose, prostate hyperplasia, osteoporosis, hyperlipidemia and hypercholesterinemia and unspecified chronic pain. A similar pattern was found for most specific services. Notably, individuals with dementia showed a similar utilization with regard to any services, but mainly received examinations, not restorative or surgical care. The same was found for patients with urinary incontinence. For outreach care, an opposite pattern was observed, with higher utilization by those with dementia and incontinence, and lower utilization by those with eye conditions, for example (Table 2).

**Table 2:** Utilization of dental services according to International Disease Classification (ICD-10, German Modification) codes by the very old in northeast Germany. Any and specific service utilization (in %) is shown

ICD-10-GM	Outpatient diagnosis code	Description	*N*	Any service	Examination	Restorative	Surgical	Prevention	Outreach
-	Total	-	404,610 (100.0)	293,766 (72.6)	275,796 (68.2)	130,227 (32.2)	131,815 (32.6)	176,881 (43.7)	54,190 (13.4)
-	UUU	Special cases, without diagnostic certainty (e.g., passing on findings or replying to health insurance enquiries or order-related services)	374,109 (92.5)	285,115 (76.2)	269,530 (72.0)	128,508 (34.4)	129,485 (34.6)	173,866 (46.5)	50,169 (13.4)
Diseases of the circulatory system	I10.90	Essential hypertension, not further described	342,446 (84.6)	258,145 (75.4)	243,736 (71.2)	115,965 (33.9)	116,838 (34.1)	157,018 (45.9)	46,122 (13.5)
Factors influencing health status and contact with health services	Z25.1*	The need for vaccination against influenza	258,991 (64.0)	208,417 (80.5)	196,138 (75.7)	94,146 (36.4)	94,386 (36.4)	126,967 (49.0)	39,824 (15.4)
Endocrine, nutritional and metabolic diseases	E11.90	Diabetes mellitus, type 2 without complications—not designated as derailed	176,727 (43.7)	131,284 (74.3)	123,542 (69.9)	55,812 (31.6)	57,858 (32.7)	76,802 (43.5)	24,379 (13.8)
Diseases of the eye and adnex	H52.4	Presbyopia	169,474 (41.9)	143,717 (84.8)	139,585 (82.4)	71,943 (42.5)	69,653 (41.1)	94,570 (55.8)	18,885 (11.1)
Diseases of the eye and adnex	H52.2	Astigmatism	161,643 (40.0)	137,038 (84.8)	133,538 (82.6)	68,915 (42.6)	66,827 (41.3)	90,658 (56.1)	16,972 (10.5)
Diseases of the circulatory system	I25.9	Chronic ischemic heart disease, not further specified	160,456 (39.7)	122,012 (76.0)	115,397 (71.9)	53,590 (33.4)	53,602 (33.4)	72,599 (45.2)	21,719 (13.5)
Diseases of the musculoskeletal system and connective tissue	M17.9	Gonarthrosis, not further described	141,200 (34.9)	114,328 (81.0)	109,212 (77.3)	54,238 (38.4)	53,792 (38.1)	71,974 (51.0)	19,323 (13.7)
Diseases of the circulatory system	I10.00	Benign essential hypertension—no indication of a hypertensive crisis	140,812 (34.8)	114,165 (81.1)	109,349 (77.7)	54,367 (38.6)	53,685 (38.1)	72,248 (51.3)	18,237 (13.0)
Diseases of the eye and adnex	H52.0	Accommodation disorders and refraction errors	139,335 (34.4)	118,870 (85.3)	116,122 (83.3)	60,546 (43.5)	58,372 (41.9)	79,292 (56.9)	13,716 (9.8)
Factors influencing health status and contact with health services	Z96.1*	Presence of an intraocular lens implant	137,658 (34.0)	116,116 (84.4)	112,464 (81.7)	55,744 (40.5)	54,654 (39.7)	74,181 (53.9)	16,767 (12.2)
Diseases of the eye and adnex	H26.9	Cataract, not further specified	134,910 (33.3)	111,938 (83.0)	108,302 (80.3)	54,962 (40.7)	53,928 (40.0)	72,375 (53.6)	15,464 (11.5)
Symptoms, signs and abnormal clinical and laboratory findings, not elsewhere classified	R32**	Unknown urinary incontinence	129,305 (32.0)	94,406 (73.0)	84,559 (65.4)	35,221 (27.2)	38,665 (29.9)	50,086 (38.7)	30,446 (23.5)
Mental and behavioral disorders	F03	Undescribed dementia	127,647 (31.5)	90,240 (70.7)	78,011 (61.1)	30,297 (23.7)	34,960 (27.4)	44,993 (35.2)	35,366 (27.7)
Symptoms, signs and abnormal clinical and laboratory findings, not elsewhere classified	R52.2**	Other chronic pain	125,881 (31.1)	102,119 (81.1)	96,357 (76.5)	47,580 (37.8)	48,388 (38.4)	63,625 (50.5)	21,517 (17.1)
Diseases of the circulatory system	I50.9	Heart failure, not further specified	118,705 (29.3)	86,459 (72.8)	79,722 (67.2)	34,410 (29.0)	36,135 (30.4)	48,242 (40.6)	21,370 (18.0)
Endocrine, nutritional and metabolic diseases	E78.5	Hyperlipidemia, not further described	114,440 (28.3)	91,462 (79.9)	87,877 (76.8)	43,435 (38.0)	42,848 (37.4)	57,953 (50.6)	13,290 (11.6)
Diseases of the musculoskeletal system and connective tissue	M16.9	Coxarthrosis, not further described	112,428 (27.8)	91,386 (81.3)	87,312 (77.7)	43,175 (38.4)	42,723 (38.0)	57,339 (51.0)	15,686 (14.0)
Endocrine, nutritional and metabolic diseases	E78.0	Pure hypercholesterolemia	101,100 (25.0)	82,035 (81.1)	79,335 (78.5)	40,860 (40.4)	39,953 (39.5)	54,098 (53.5)	11,094 (11.0)
Diseases of the circulatory system	I70.9	Generalized and unspecified atherosclerosis	95,316 (23.6)	76,406 (80.2)	72,509 (76.1)	34,732 (36.4)	34,715 (36.4)	46,424 (48.7)	13,296 (13.9)
Diseases of the musculoskeletal system and connective tissue	M81.99	Osteoporosis, not further described—not further described Localization	95,150 (23.5)	76,401 (80.3)	72,311 (76.0)	34,883 (36.7)	34,424 (36.2)	46,755 (49.1)	14,847 (15.6)
Factors influencing health status and contact with health services	Z92.1*	Long-term therapy (present) with anticoagulants in the patient's own medical history	89,815 (22.2)	72,965 (81.2)	70,386 (78.4)	34,552 (38.5)	33,519 (37.3)	46,107 (51.3)	10,997 (12.2)
Diseases of the circulatory system	I83.9	Varices of the lower extremities without ulceration or inflammation	88,616 (21.9)	72,138 (81.4)	69,386 (78.3)	34,830 (39.3)	33,918 (38.3)	45,946 (51.8)	10,932 (12.3)
Endocrine, nutritional and metabolic diseases	E79.0	Hyperuricemia without signs of inflammatory arthritis or tophic gout	77,737 (19.2)	59,947 (77.1)	57,278 (73.7)	27,528 (35.4)	27,897 (35.9)	37,144 (47.8)	9837 (12.7)
Diseases of the genitourinary system	N40	Prostatic hyperplasia	74,931 (18.5)	61,865 (82.6)	60,019 (80.1)	31,427 (41.9)	30,526 (40.7)	40,871 (54.5)	7528 (10.0)

*Categories Z00-Z99 are intended for cases in which facts are indicated as “diagnoses” or “problems” which cannot be classified as disease, injury or external cause under categories A00-Y89

**This chapter includes (subjective and objective) symptoms, abnormal results of clinical or other investigations, and inaccurately identified conditions for which there is no classifiable diagnosis elsewhere

We further assessed the utilization of any dental service stratified according to different GM-DRGs (Table 3). Utilization of any service was higher in participants hospitalized for non-severe gastrointestinal ulcerations, non-severe arrhythmia, bronchitis, non-severe hypertension, syncope, non-severe renal insufficiency, and non-complicated cardial diagnostics or eye operations. Utilization was lower in patients with severe gastrointestinal ulcerations as well as severe heart insufficiency. These trends of higher or lower utilization were similar for other services, except outreach care, where a different pattern emerged: utilization was higher in patients with non-severe but also severe ulcerations, paraplegia/tetraplegia, non-severe hypertension, infections, head or skin injuries, joint operations, apoplexy, and geriatric rehabilitation. It was lower in patients with bronchitis (Table 3).

**Table 3:** Utilization of dental services according to German modified diagnosis-related groups (GM-DRGs). Any and specific service utilization (*N*; %) is shown

GM-DRG	Description	*N*	Any service	Examination	Restorative	Surgical	Prevention	Outreach
-	Total population	404,610 (100.0)	293,766 (72.6)	275,796 (68.2)	130,227 (32.2)	131,815 (32.6)	176,881 (43.7)	54,190 (13.4)
F62B	Cardiac insufficiency and shock with extremely serious complications or comorbidity, with dialysis or complicated diagnosis or with certain high-level treatment or without complicated constellation, without specific high-level treatment, more than 1 day of occupancy in certain acute renal failure with extremely severe complications or comorbidity	40,295 (10.0)	27,904 (69.2)	25,828 (64.1)	10,013 (24.8)	10,614 (26.3)	14,387 (35.7)	6928 (17.2)
G67C	Esophagitis, gastroenteritis, gastrointestinal hemorrhage, ulcer disease and various diseases of the digestive organs without certain or other complicating factors, without extremely severe complications or comorbidity	21,650 (5.4)	17,218 (79.5)	16,094 (74.3)	7150 (33.0)	7536 (34.8)	9712 (44.9)	4170 (19.3)
I41Z	Geriatric early rehabilitative complex treatment for diseases and disorders of the musculoskeletal system and connective tissue	21,131 (5.2)	16,507 (78.1)	15,388 (72.8)	6925 (32.8)	7469 (35.3)	9760 (46.2)	4835 (22.9)
K62B	Various metabolic diseases in paraplegia/tetraplegia or with complicated diagnosis or endoscopic insertion of a gastric balloon or age < 16 years, one occupancy day or without extremely severe complications or comorbidity or without certain costly/highly complex treatment	19,998 (4.9)	15,445 (77.2)	14,251 (71.3)	6289 (31.4)	6565 (32.8)	8721 (43.6)	4045 (20.2)
G67B	Esophagitis, gastroenteritis, gastrointestinal bleeding, ulcer disease, and various diseases of the digestive organs with other complicating factors or with extremely severe complications or comorbidity	19,637 (4.9)	14,148 (72.0)	12,582 (64.1)	4864 (24.8)	5555 (28.3)	7112 (36.2)	4998 (25.5)
F71B	Non-severe cardiac arrhythmias and conduction disturbances without extremely severe complications or comorbidity or occupancy day, without catheter-assisted electrophysiological examination of the heart, without specific high-level treatment	16,666 (4.1)	13,869 (83.2)	13,453 (80.7)	6919 (41.5)	6635 (39.8)	9023 (54.1)	1930 (11.6)
F67D	Hypertension without complicated diagnosis, without extremely severe or severe complications or comorbidity, without certain moderately complex/complicated treatment, age > 17 years	15,300 (3.8)	12,722 (83.2)	12,361 (80.8)	5990 (39.2)	5932 (38.8)	7833 (51.2)	1825 (11.9)
E77I	Infections and inflammation of the respiratory system without complex diagnosis, without extremely severe complication or comorbidity or a complication or comorbidity, age > 0 years, except for para/quadriplegia, without complex treatment in multidrug-resistant pathogens	15,143 (3.7)	11,288 (74.5)	10,023 (66.2)	3889 (25.7)	4397 (29.0)	5701 (37.6)	3847 (25.4)
F48Z	Geriatric early rehabilitative complex treatment for diseases and disorders of the circulatory system	14,512 (3.6)	10,620 (73.2)	9894 (68.2)	4084 (28.1)	4401 (30.3)	5924 (40.8)	2867 (19.8)
L63F	Infections of the urinary organs without extremely severe complications or comorbidity, without certain moderately costly/elaborate/highly costly treatment, without complex treatment multi-resistant pathogens (MRE), without certain serious infections, age > 5 and < 18 years, without severe complications or comorbidity or age > 17 and < 90 years	13,704 (3.4)	10,259 (74.9)	9171 (66.9)	3754 (27.4)	4170 (30.4)	5338 (39.0)	3521 (25.7)
I68D	Non-surgically treated diseases and injuries of the spinal column, more than one occupancy day or other femoral fracture, without sacrum fracture, without certain moderately elaborate, elaborate or highly elaborate treatment	12,645 (3.1)	9936 (78.6)	9328 (73.8)	4016 (31.8)	4269 (33.8)	5590 (44.2)	2356 (18.6)
F73Z	Syncope and collapse	12,352 (3.1)	10,638 (86.1)	10,328 (83.6)	5144 (41.6)	5053 (40.9)	6745 (54.6)	1754 (14.2)
B80Z	Other head injuries	11,645 (2.9)	9202 (79.0)	8302 (71.3)	3502 (30.1)	3861 (33.2)	4939 (42.4)	3293 (28.3)
L60D	Renal insufficiency, more than one occupancy day, without dialysis, without extremely severe complications or comorbidity, age > 17 years or without severe complications or comorbidity, without complex intensive care treatment > 196/184/- expense points	10,975 (2.7)	9208 (83.9)	8803 (80.2)	4535 (41.3)	4471 (40.7)	5977 (54.5)	1707 (15.6)
I47B	Revision or replacement of the hip joint without certain complicated factors, with complex diagnosis of the pelvis/thigh, with certain endoprosthetic or joint plastic surgery of the hip joint, with implantation or replacement of a radius head prosthesis	10,969 (2.7)	7827 (71.4)	7084 (64.6)	2826 (25.8)	3141 (28.6)	4150 (37.8)	2497 (22.8)
J65Z	Injury of the skin, subcutis, and mamma	10,541 (2.6)	8112 (77.0)	7420 (70.4)	3033 (28.8)	3282 (31.1)	4321 (41.0)	2701 (25.6)
E65C	Chronic obstructive pulmonary disease without extremely severe complication or comorbidity, without complicated diagnosis, without FEV1 < 35% or a complication or comorbidity, age > 1 year, without specific moderately complex/expensive treatment	10,058 (2.5)	7425 (73.8)	6946 (69.1)	2616 (26.0)	2874 (28.6)	3727 (37.1)	1628 (16.2)
C08B	Extracapsular extraction of the lens (ECCE) without congenital malformation of the lens or certain interventions on the lens	9831 (2.4)	8211 (83.5)	7957 (80.9)	3974 (40.4)	4037 (41.1)	5311 (54.0)	1396 (14.2)
A90A	Partial stationary geriatric complex treatment	9830 (2.4)	7470 (76.0)	6755 (68.7)	2749 (28.0)	2991 (30.4)	3825 (38.9)	2243 (22.8)
E69B	Bronchitis and bronchial asthma, more than 1 day of treatment age > 55 years or with extremely severe or severe complication or comorbidity, age > 0 years or 1 day of treatment or without extremely severe or severe complication or comorbidity, age < 1 year or flexible bronchoscopy, age < 16 years or determined moderate treatment, with RS virus infection	9777 (2.4)	8731 (89.3)	8674 (88.7)	5001 (51.2)	4662 (47.7)	6247 (63.9)	415 (4.2)
F49G	Invasive cardiological diagnosis except in acute myocardial infarction, without extremely severe complication or comorbidity, age > 17 years, without cardiac mapping, without severe complication or comorbidity at day of treatment > 1, without complex diagnosis, without specific intervention	9574 (2.4)	7852 (82.0)	7529 (78.6)	3719 (38.8)	3860 (40.3)	5003 (52.3)	1431 (14.9)
I34Z	Geriatric early rehabilitative complex treatment with specific operating room procedure for diseases and disorders of the musculoskeletal system and connective tissue	9419 (2.3)	7294 (77.4)	6778 (72.0)	2935 (31.2)	3191 (33.9)	4155 (44.1)	2106 (22.4)
F62D	Cardiac insufficiency and shock without extremely serious complications or comorbidity or without dialysis, without complicated diagnosis, without complicated constellation, without specific high-level treatment, 1 day of occupancy	8615 (2.1)	6969 (80.9)	6719 (78.0)	3050 (35.4)	3180 (36.9)	4033 (46.8)	1292 (15.0)
B70B	Apoplexy with neurological complex treatment of acute stroke, more than 72 h, without complicated diagnosis or with complex cerebrovascular vasospasm or intensive care complex treatment	8499 (2.1)	6391 (75.2)	5850 (68.8)	2582 (30.4)	2754 (32.4)	3672 (43.2)	1883 (22.2)
L64A	Other urinary organ diseases with extremely severe or severe complications or comorbidity or certain diagnosis, more than one occupancy day or urethra-cystoscopy, congenital malformation or age < 3 years	5669 (1.4)	4283 (75.6)	3986 (70.3)	1778 (31.4)	1820 (32.1)	2426 (42.8)	1082 (9.1)

In multi-variable analysis, social hardship status (OR: 1.14; 95% CI: 1.12–1.16), federal state (Brandenburg 0.85; 0.84–0.87; Mecklenburg-Western Pomerania: 0.80; 0.78–0.82) and age significantly affected utilization (0.95; 0.95–0.95/year), together with a range of co-morbidities according to ICD-10 and GM-DGRs (Table 4, Table S1). Pseudo-*R*^2^ indicated that the model generally had limited explanatory power (*R*^2^ = 0.15).

**Table 4:** Multivariable analysis of factors associated with utilization of any dental service. We here show abbreviated results, excluding the association with general medical conditions (the full results can be found in the Appendix)

Covariate	OR	2.5%	97.5%	*p* value
Sex (male)	0.982	0.964	1.002	0.071
Social hardship status (yes)	1.137	1.118	1.157	< 0.001
Deceased (yes)	0.149	0.144	0.153	< 0.001
Federal state (Brandenburg)	0.853	0.837	0.870	< 0.001
Federal State (Mecklenburg-Western Pomerania)	0.798	0.781	0.815	< 0.001
Age (year)	0.946	0.945	0.948	< 0.001

## Discussion

Understanding dental service utilization in specific populations and groups may allow to increase access to the right services for every individual, thereby improving health and services’ efficiency and equitability [13]. The present study tried to evaluate how factors driving services’ needs (age, sex, general health) and access on patient level (income and financial means, place of living) and system level (physical and organizational) impact on utilization [14, 15]. We hypothesized that the utilization of dental services in the very old was associated with an individual’s age, general health status, place of living, and social status. Moreover, we assumed to find service-specific disparities. We confirm these hypotheses; social, demographic, regional, and general health aspects were associated with the utilization of dental services in very old Germans.

A number of aspects should be discussed. First, utilization in this specific group was comparably high; in general, dental service utilization in Germany is higher than that in most other countries, likely due to the setup of the service provision, with most services being available at no costs at all to the patient [16]. Moreover, regular consumption of dental services is incentivized using a bonus scheme, with patients getting a discount on their out-of-pocket expenses for prosthetic services in case they can demonstrate a history of regular yearly checkups. Such incentive will be especially efficacious in old individuals, who either have or expect to have prosthetic services with higher likelihood than younger ones.

We also found only minimal changes in the age-specific utilization over the 6-year period; that is, seniors of similar age did not show considerably increased utilizations in 2012 compared with 2017, for example. The only detectable increase occurred between 2012 and 2013, most likely associated with a general policy shift in dental healthcare in Germany (an entry fee existing until 2012, with patients paying 10 Euro to the practice—which passed it on to the insurer—whenever entering the practice for the first time in a quarter of a year had been abolished in 2013). These findings of rather constant utilization over the first half of the last decade as well as the increase in utilization of dental services from 2012 and 2013 are in line with previous research [17, 18]. Our findings are in so far relevant, as a number of major policy shifts targeting the very old requiring care assistance have been introduced between 2013 and 2015, the effects of which our analysis did not capture (so far). This might be as we only included individuals aged 75 years or older in 2012 and followed them for 6 years (i.e., those entering this group later on were not included), but also as we did not focus on those requiring care assistance, i.e., probably “diluted” their relevance in our analysis. It would be relevant to re-assess this cohort, expanding it to individuals aged 75 years or older in 2018 and focusing on only those receiving care assistance**.**

We find a drastic and only limitedly service-specific decrease in utilization with age; individuals aged 85 years, for example, consumed only a fraction of services compared with those age 75 years. Notably, from age 95 years onwards utilization was fairly stable, indicating a possible “survivor” effect. The only exception from these observations was outreach care, as discussed below. Age is associated with an increasing prevalence of chronic and severe diseases or hospitalization [19]. In line with our previous analysis on prosthetic services, such severe general health conditions (e.g., severe gastrointestinal ulcerations as well as severe heart insufficiency) were found to significantly decrease utilization. Notably, for most other (especially ICD-10 coded) conditions, the overall utilization was unaffected. This might be as ICD-10 codes were derived from ambulatory assessments, where individuals need to attend their general practitioners and hence show some kind of mobility and self-capability. Moreover, individuals with dementia (and incontinency) showed reduced utilization of therapeutic services (but not examinations). This might be as these individuals do not accept more intense (and time consuming) care for treatment.

We further assessed the impact of social hardship status on utilization. Such status is a proxy for low income. It has been found associated with increased utilization of prosthetic services, as individuals with this status usually pay very low or no additional fee at all for any prosthetic service; that is, financial utilization barriers for this type of dental treatment are very low or absent [7]. For the present analysis, hardship status was used only as a social marker, as the analyzed dental services (examination, restorations, surgery, prevention, outreach care) are coming at no costs for all statutorily insured individuals, regardless of their age. This is a remarkable difference of the German compared with many other healthcare systems, where retirement oftentimes means loss of professionally supported health insurance [20–22] and a subsequent collapse of service utilization [23]. It is noteworthy that utilization for those with hardship status was found significantly increased in multivariable analysis (in bivariate analyses this was less clear, indicating possible confounding by age, place of living, or health status, for example). As those with low social status are also likely to show the poorest oral and general health [24], it is highly relevant to find them to consume services more often, too. It is beyond this study to elucidate the reasons underlying this utilization. Notably, though, existing public policies to support healthcare utilization in vulnerable groups in Germany, e.g., those with chronic diseases [25], do not capture those with economic constraints and poor oral health, i.e. cannot be at the heart of this association. Independently of the found increased utilization, policy makers may want to revisit such policies and to strengthen dental service utilization for the very old, the very sick, and the very poor.

We also found an association between utilization and place of living [13]. Such association has been assumed to be grounded in rural areas being underserviced due to workforce shortages while urban areas suffer from provider clustering and associated supply-side-induced demand [26, 27]. We confirm such rural-urban disparities for any service utilization in the very old. The two rural federal states in our study, Brandenburg and Mecklenburg-Western Pomerania, show much lower dentist densities than Berlin [28], possibly explaining our findings. Notably, utilization in the whole population (not only the very old) has been found to follow the opposite pattern, with higher utilization in the two rural states than in Berlin [17]. Hence, the observed inequalities seem to be moderated by age: older individuals seem to seek care more often, but are not able to physically access it in rural areas, while younger individuals could access it more easily in urban areas, but are not seeking care. We want to highlight that our analyses on smaller spatial level (municipalities) showed a more nuanced picture, with some rural areas showing high utilization of specific (but not all) services. We are so far unable to entangle possible reasons underlying this observation, which may be grounded in local dentist densities (some municipalities show surprisingly high densities) or a locally increased proportion of dentists with specific contractual agreements with care homes (thereby increasing access to care for the very old). More in-depth analyses seem warranted to first confirm and then explain such peculiar patterns, as they may allow to identify local best practices which could be translated to regional or national level.

We identified service-specific utilization patterns not only across regions, as described, but also age. Our findings of a generally decreasing utilization between age 75 years and 95 years have been identified before, with utilization of dental therapeutic services decreasing by around 50% along this age span in a national sample [17]. In the national sample, restorative care was provided far more often than surgical care, while we found restorative care being consumed to a similar degree like surgical care. This might be as our sample was generally older and also represented a different target population (see below). We assume that these two factors drive a treatment concept where maintaining teeth (using restorative care) is deprioritized while achieving an overall pain free status (by removing teeth, for example) is getting more important (and usually also being the only available option). Notably, prevention (which was only calculus removal in the present study) continued to be provided up to high age (albeit to a lower intensity).

The only service where utilization was increasing with age was outreach care, while this seemed to allow for only very limited provision of therapies. It is relevant to understand the drivers behind treatment patterns in outreach care, and it may not be sufficient to only incentivize outreach visits, but also support outreach management or referral concepts for those requiring more complex care. In light of the Covid-19 pandemic and the near-global shutdown of any dental visits (except for emergencies) to care homes (also in Germany), outreach care is likely to be re-evaluated with regard to its benefits and risks.

Overall, our study calls for a range of possible policy and research initiatives: First, healthcare policy and decision makers should install incentives to provide services to the high needs elderly population. This may come by increasing single treatment fees for this group, or more generally by making outreach services more attractive. The latter may be realized by increasing fees once more or trialing and allowing different kinds of servicing, e.g., involving task delegation to assistance personnel. Outreach care should further be provided not only to individuals in long-term care centers (nursing homes) but also to those residing at home (which is the vast majority of elderly). Similarly, referral schemes between general medical practitioners and dentists may be helpful to identify high-risk individuals; mandatory follow-ups after such referrals may make sure that sick and remote older individuals (who seldom proactively seek care) are not plainly overlooked by standard dental healthcare. Integrated service models (for example oral and dental hygiene enforcement for patients at risk for pneumonia) should further be strengthened. Dental research, on the other hand, is called to action to develop applicable concepts encompassing effective management of dental diseases at optimal infection and transmission control measures. Right now, servicing is at a minimal level due to fears of infection and it can be expected that infection control will remain a highly relevant topic in this vulnerable population even when Covid-19 is finally brought behind us. Moreover, dental research should develop and evaluate the described complex care models involving delegation or cooperation. A number of initiatives are currently underway in Germany in this direction (e.g., https://innovationsfonds.g-ba.de). Further, primary and secondary prevention models in this group should be enhanced; currently prevention concepts in the elderly are by large identical with those in younger individuals. Policy makers may want to revisit such age-group-specific prevention concepts when they are available. Generally, we see a great need to emphasize prevention in this group (based on our findings, prevention was near-absent for the very old in the Northeast). Dentists and dental bodies may want to actively participate in such research and also the implementation of possible policies, especially considering that with the very old, there is a growing group with high needs who can truly benefit from dental care.

This study has a number of strengths and limitations. First, this is one of few longitudinal studies evaluating dental service utilization in very old individuals. Our cohort involved over 400,000 individuals from three federal states spanning an area of similar size as Austria or the Netherlands and Belgium combined. Second, we evaluated a range of demographic, social, general health, and regional factors, some of which (DRGs, ICD-10) have not routinely been employed when evaluating dental healthcare. Third, and as a limitation discussed above, claims data suffer from a range of biases. Provided and claimed treatment cannot be equated with needs or morbidity. Exploring causality is only limitedly possible, and within the present (largely descriptive) analyses, this was also not within our scope (the available longitudinal data may permit some more in-depth analyses in the future). Any identified bivariate association may suffer from confounding bias, and even the performed multivariable analysis showed only limited explanatory value, likely as further relevant factors (e.g., medication, care status) were not available and accounted for, or as available factors (e.g., social hardship status, place of living) came with very limited granularity. Fourth, individuals insured by AOK Nordost are not fully representative for other individuals from the same target area or even the whole of Germany: more affluent people are often not statutorily insured (there is a minimum income level defined as entry barrier into private insurances in Germany). This may affect the individual’s health status and his or her utilization behavior (reflecting health literacy, but also specific incentives set by insurers towards seeking or avoiding care) as well as the number and type of services provided by the dentists (as services are remunerated differently). The Northeast of Germany is over-proportionally old and, as mentioned, economically comparably weak (notably, there is a significant economic disparity within the Northeast, too, which our data reflect on). The rural parts of the Northeast suffered from emigration to other areas of Germany especially after the reunification, while Berlin experienced an over-proportional immigration in the 1960s from aboard as well as the last 20 years from within Germany. These specifics will impact service utilization but may not be found to this degree in other areas of Germany. Future studies on the present dataset may explore them in detail, if possible, to better understand what impact on utilization they have.

In conclusion, and within the limitations of this study, social, demographic, regional, and general health aspects were associated with the utilization of dental services in very old Germans. We identified consistent and considerable disparities in utilization between populations. Policies to allow service utilization also for the sick, economically disadvantaged, rural, and very old should be developed, tested, and employed.

## Electronic supplementary material

ESM 1 (XLSX 9.20 kb)

## Data Availability

Data used in this study cannot be made available by the authors given data protection rules, but may be requested at the GEWiNO.
